# Protection of prior SARS-CoV-2 infection, COVID-19 boosters, and hybrid immunity against Omicron severe illness: A population-based cohort study of five million residents in Canada

**DOI:** 10.1371/journal.pone.0299304

**Published:** 2024-02-23

**Authors:** Shishi Wu, Yanhong Li, Stefan Baral, Sharmistha Mishra, Maria Koh, Haley Golding, Jeffrey C. Kwong, Xiaolin Wei

**Affiliations:** 1 Dalla Lana School of Public Health, University of Toronto, Toronto, Ontario, Canada; 2 Department of Epidemiology, Johns Hopkins University, Baltimore, Maryland, United States of America; 3 MAP Centre for Urban Health Solutions, St. Michael’s Hospital, Toronto, Ontario, Canada; 4 Institute of Health Policy, Management and Evaluation, University of Toronto, Toronto, Ontario, Canada; 5 Department of Medicine and Institute of Medical Sciences, University of Toronto, Toronto, Ontario, Canada; 6 Institute for Clinical Evaluative Sciences, Toronto, Ontario, Canada; 7 Public Health Ontario, Toronto, Ontario, Canada; 8 Centre for Vaccine Preventable Diseases, University of Toronto, Toronto, Ontario, Canada; 9 Department of Family and Community Medicine, University of Toronto, Toronto, Ontario, Canada; 10 University Health Network, Toronto, Ontario, Canada; Alexandria Medicine: Alexandria University Faculty of Medicine, EGYPT

## Abstract

**Background:**

Evidence on protection of different patterns of infection- and vaccine-acquired immunity against Omicron-associated severe illness is useful in planning booster vaccination strategies. We examined protection of prior SARS-CoV-2 infection, a third or a fourth COVID-19 vaccine dose, and hybrid immunity against Omicron-associated severe illness.

**Methods and findings:**

This population-based cohort study followed five million individuals with at least one SARS-CoV-2 RT-PCR test before November 21, 2021 until an Omicron-associatedhospitalization or death. We used Cox regression models to estimate risks of Omicron-associated hospitalization and a composite severe outcome (hospitalized and death), among individuals with infection- and/or vaccination-acquired immunity. Individuals who were unvaccinated and had no history of a prior infection severed as the reference group. Both adjusted hazard ratios (HR) and corresponding protection (one minus adjusted HR), with 95% confidence intervals (CIs), were reported. Three doses provided 94% (95%CI 93–95) and 93% (95%CI 91–94) protection against Omicron-associated hospitalization at 2–3 and ≥3 months post-vaccination respectively, similar to the protection conferred by three doses and a prior infection (2–3 months: 99%, 95%CI 97–100; ≥3 months: 97%, 95%CI 92–99) and four doses (1 month: 87%, 95%CI 79–92; 1–2 months: 96%, 95%CI 92–98). In individuals ≥65 years old, protection of four doses increased to 95% (95%CI 91–98) at 1–2 months, significantly higher than that of three doses over the follow-up period. Similar results were observed with the composite severe outcome.

**Conclusion:**

At least three antigenic exposures, achieved by vaccination or infection, confers significant protection against Omicron-associated hospitalization and death in all age groups. Our findings support a third dose for the overall population, regardless of prior infection status, and a fourth dose for the elderly to maintain high level of immunity and substantially reduce risk of severe illness at individual level.

## Introduction

The high level of immunological protection conferred by past SARS-CoV-2 infections and COVID-19 vaccination in preventing severe COVID-19-related illness helps preserve health system resources and saves millions of lives globally [1]. Pre-Omicron, both infection-acquired and vaccination-induced immunity showed over 90% protection against COVID-19-related hospitalization [2–5]. Hybrid immunity, defined as immunity acquired from both prior infection and vaccination, was reported to reduce risk of pre-Omicron hospitalization or fatal reinfection by over 70% [6].

The Omicron variant, first identified in November 2021, has spread rapidly becoming the dominant variant worldwide due to its immune-evasive properties. Although data show substantially reduced protection of both infection-acquired and vaccination-induced immunity against Omicron infections [7–12], protection against severe illness seems to be preserved. Despite a recent study observing lower protection against hospitalization (28·5%) [13], early observational studies reported 70–90% effectiveness of a prior non-Omicron infection in preventing Omicron-associated hospitalization and death [7, 8, 14, 15]. In terms of vaccine-induced and hybrid immunity, two vaccine doses maintain 70–80% effectiveness against Omicron-associated severe illness for over 4 months [16–19], and early data suggests that a third dose or having a prior infection among individuals with two doses can boost protection to over 90% against hospitalization or death [7–9, 14]. Recognizing the waning immunity following COVID-19 vaccines, high-income countries began administering a third dose (the first booster) in mid-2021. Subsequently, towards the end of 2021, a fourth dose, or the second booster, was introduced, targeting high-risk population groups in response to the emerging challenge posed by the Omicron variant, known for its partial immune evasion. As the Omicron wave continues, understanding the effectiveness of different patterns of immunity acquired from previous SARS-CoV-2 infections and vaccines can help anticipate health-care burden and make plans for booster vaccination strategies. Therefore, evidence on how a past SARS-CoV-2 infection modifies the protection conferred by a third and a fourth dose in preventing severe outcomes associated with Omicron infection and how hybrid immunity compares to four vaccine doses is needed but currently scarce.

Our study aims to evaluate the protective effects conferred by immunity from prior infection and to elucidate how this immunity interacts with the protection provided by a third and a fourth dose of COVID-19 vaccines in preventing specific severe outcomes—namely hospitalization, death, and a composite of these events—associated with the Omicron variant. Moreover, we seek to analyze the levels of protection over time, particularly examining the durability of immunity from the time since the last vaccine dose was administered.

## Methods

### Study participants, design, and data sources

This study is a follow up study that examined the incremental protection of previous infection against Omicron infection, which was published previously [20]. Among 14·9 million Ontario residents covered by the Ontario Health Insurance Plan (OHIP), we selected individuals who had at least one real-time polymerase chain reaction (RT-PCR) test for SARS-CoV-2 between January 15, 2020 and November 21, 2021. We excluded individuals who did not have any RT-PCR test during this period, individuals who were less than 12 years old, individuals who died before November 22, 2021, and those who were long-term care residents or immunocompromised. Each individual was followed from November 22, 2021, when the first Omicron case was identified in Ontario, until the end of the study (March 21, 2022) or the primary outcomes–hospitalization or death associated with Omicron infection–were observed. We ended follow-up on March 21, 2022 to ensure only one disease episode of Omicron infection would occur during the follow-up window. During the follow-up window, Omicron BA.1 and BA.2 were the predominant variants circulating in Ontario.

We extracted all RT-PCR testing data from the Ontario Laboratories Information System (OLIS), Distributed Labs, and the Public Health Case and Contact Management Solution (CCM) databases. To ascertain baseline information on socio-demographic and geographic characteristics and chronic conditions, we linked our study cohort to population-based provincial health administrative databases. Additionally, we retrieved COVID-19 vaccination data, including dates of administration and dose number, from COVaxON, a centralized COVID-19 vaccine information system. These datasets were linked using unique encoded identifiers and analyzed at ICES (formerly the Institute for Clinical Evaluative Sciences).

### Exposure

The main exposure was a prior SARS-CoV-2 infection, which was determined at the baseline by any positive test at least 90 days before an Omicron infection. Individuals without any positive tests and with at least one negative test since January 2020 were classified as unexposed.

In Ontario, the COVID-19 vaccination campaign commenced in December 2020. By July of the following year, all residents 12 years and older became eligible to receive the vaccine. The administration of third doses began in August 2021, initially targeting immunocompromised individuals and long-term care residents. All individuals aged ≥70 years and healthcare workers became eligible on November 6, followed by individuals aged ≥50 years on December 13 and individuals aged ≥18 years on December 18 [19]. Ontario started offering a fourth dose to high-risk populations, including immunocompromised individuals, long-term care and retirement home residents, and older adults in congregate settings who had received a third dose at least three months previously, in December, 2021. This eligibility was extended to include all adults in July 2022. BNT162b2 (Pfizer-BioNTech Comirnaty), mRNA-1273 (Moderna Spikevax), and ChAdOx1 (Astra-Zeneca Vaxzevria, COVIDSHIELD) were the primary COVID-19 vaccines provided in Ontario [19]. BNT162b2 (Pfizer-BioNTech Comirnaty) and mRNA-1273 (Moderna Spikevax) were approved as booster doses. Vaccination status was assessed based on the number of vaccine doses an individual had received prior to an Omicron infection, without differentiating between vaccine products. An individual’s vaccination status was considered updated 14 days following the administration of the first or second dose, and 7 days after the administration of the third or fourth dose. Vaccination status was treated as a time-varying variable during follow-up. Our analysis was stratified by time since vaccination (<1 month, 1–2 months, 2–3 months, ≥3 months).

### Outcomes

The main outcomes were hospitalization and death associated with an Omicron infection. Infection with the Omicron variant was identified by whole genome sequencing (WGS) as the B.1.1.529 lineage or having S-gene target failure (SGTF) during the study period. SGTF is a reliable proxy for Omicron identification, with 98·9% sensitivity and 99·9% specificity during December 2021 and January 2022 in Ontario [21]. We identified these outcomes using CCM (for both hospitalization and death), the Canadian Institute for Health information’s Discharge Abstract Database (for hospitalization), and the Ontario Registered Persons Database (for death). For Omicron-associated hospitalization identified using the Discharge Abstract Database, a positive RT-PCR test result must have occurred within 15 days before or three days after admission. For Omicron-associated death identified using the Ontario Registered Persons Database, a positive test result must have occurred within 30 days before death. We created a composite severe outcome consisting of all Omicron-associated hospitalization and death cases.

### Covariates

We extracted patients’ demographic and residential information, including age, sex, rural residence, and long-term care facility residence. From 2016 Canadian census data, we obtained area-level information on socioeconomic status, such as household income quintile and proportion of the working population employed as non-health essential workers [22]. We identified individuals’ pre-existing comorbidities using health administrative databases and existing ICES chronic disease cohorts that were created using validated algorithms that have high sensitivity and specificity (S2 Table in S1 Appendix). The number of comorbidities was assigned to each individual. Comprehensive definitions of all variables and the databases they were sourced from are listed in S1, S2 Tables in S1 Appendix.

### Statistical analysis

Following descriptive analysis, we estimated the hazard ratio for Omicron-associated hospitalization, death, and the composite severe outcome using Cox proportional hazards regression models by comparing the risks of these Omicron-associated severe outcomes among individuals with infection- and/or vaccination-acquired immunity to those with no immunity at different time intervals (<1 month, 1–2 months, 2–3 months, and ≥3 months). Individuals who were unvaccinated and had no history of a prior infection severed as the reference group in all the models. The analyses for the three outcomes were further stratified by age (≤64 years old and ≥65 years old). We adjusted for all listed covariates in all models. Both adjusted hazard ratios (HR) and corresponding protection (one minus adjusted HR), with 95% confidence intervals (CIs), were reported. For imputing missing data, we used the median for continuous variables and a random imputation method based on the distribution of the non-missing values for categorical variables. The Cox model proportionality assumptions were verified using Schoenfeld residuals.

We conducted all analyses in R version 3.6.1 (R foundation for statistical computing, Vienna, Austria) between May and October 2022 using de-identified data. The findings were reported in accordance with the STROBE guidelines for cohort studies (S2 Section in S1 Appendix). All tests were two-sided and used p<0.05 as the level of statistical significance [20].

### Ethics approval

ICES is a prescribed entity under Ontario’s Personal Health Information Protection Act (PHIPA). Section 45 of PHIPA authorizes ICES to collect personal health information, without consent, for the purpose of analysis or compiling statistical information with respect to the management of, evaluation or monitoring of, the allocation of resources to or planning for all or part of the health system. Projects that use data collected by ICES under section 45 of PHIPA, and use no other data, are exempt from REB review. The use of the data in this project is authorized under section 45 and approved by ICES’ Privacy and Legal Office.

## Results

**Fig 1:**
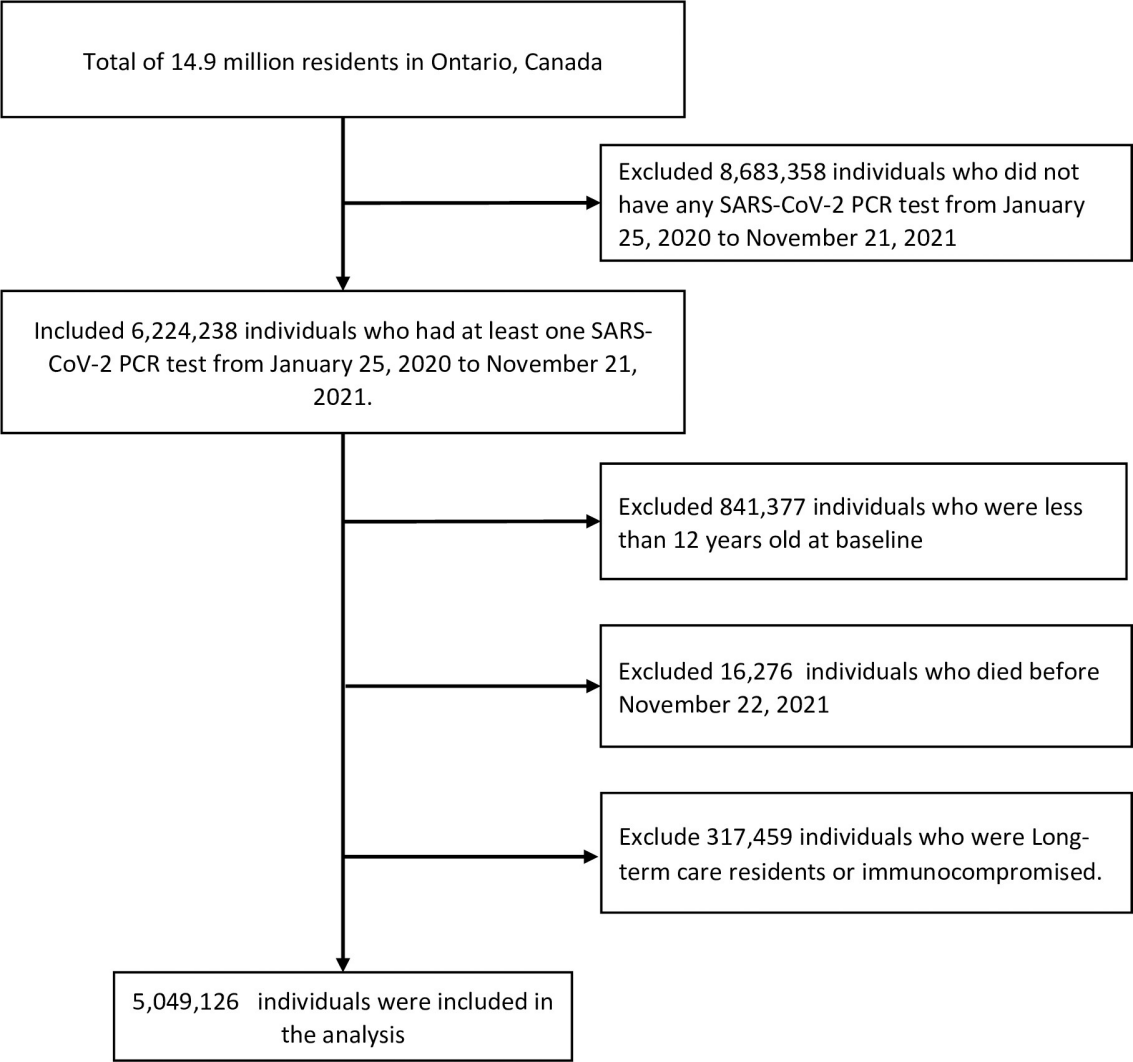
Flow chart of participants in the cohort.

We excluded 16,276 participants who died before the start of follow-up and 841,377 aged <12 years. We also excluded a total of 317,459 long-term care residents and immunocompromised individuals. After applying the exclusion criteria, we identified over five million eligible individuals. Over five million eligible Ontario residents were included in the cohort (Fig 1), among which 464,665 had a prior RT-PCR confirmed SARS-CoV-2 infection and 4,584,461 without a documented positive result were considered infection-naïve. Compared to the infection-naïve group, previously infected individuals were more likely to be working-age adults, male, have no comorbidities, and reside in areas with lower household income and higher proportions of essential workers. As of November 22, 2021, 83·7% of the cohort was vaccinated with two doses; 2·6% was vaccinated with a third or a fourth dose. Only 10·8% of the cohort remained unvaccinated (Table 1). The full list of demographic characteristics is given in S3 Table in S1 Appendix.

**Table 1:** Demographic characteristics and vaccination status at baseline^a^.

Characteristics	Prior infection		Total individuals (N = 5,049,126)
	No (n = 4,584,461)	Yes (n = 464,665)	
Age group, N (%) (years)			
12–19	477508 (10·4)	54447 (11·7)	531955 (10·5)
–29	812737 (17·7)	100257 (21·6)	912994 (18·1)
30–39	825057 (18·0)	87076 (18·7)	912133 (18·1)
40–49	703236 (15·3)	79538 (17·1)	782774 (15·5)
50–59	729788 (15·9)	76263 (16·4)	806051 (16·0)
60–69	549152 (12·0)	42133 (9·1)	591285 (11·7)
70–79	308033 (6·7)	16927 (3·6)	324960 (6·4)
≥80	178950 (3·9)	8024 (1·7)	186974 (3·7)
Sex, N (%)			
Male	2103205 (45·9)	229780 (49·5)	2332985 (46·2)
Female	2481256 (54·1)	234885 (50·5)	2716141 (53·8)
Rural resident, N (%)			
No	4112660 (89·7)	443062 (95·4)	4555722 (90·2)
Yes	460589 (10·0)	20453 (4·4)	481042 (9·5)
Neighborhood income quintile ^b^			
1 (lowest)	845098 (18·4)	111649 (24·0)	956747 (18·9)
2	873416 (19·1)	98819 (21·3)	972235 (19·3)
3	916082 (20·0)	99546 (21·4)	1015628 (20·1)
4	945846 (20·6)	84491 (18·2)	1030337 (20·4)
5 (highest)	991010 (21·6)	68812 (14·8)	1059822 (21·0)
Number of comorbidities, N (%)			
0	2561392 (55·9)	282177 (60·7)	2843569 (56·3)
1	1242167 (27·1)	120313 (25·9)	1362480 (27·0)
2	448104 (9·8)	39365 (8·5)	487469 (9·7)
≥3	332798 (7·3)	22810 (4·9)	355608 (7·0)
Essential workers quintile ^b^			
1 (0%–32·5%)	1019286 (22·2)	72616 (15·6)	1091902 (21·6)
2 (32·5%–42·3%)	1032954 (22·5)	97692 (21·0)	1130646 (22·4)
3 (42·3%–49·8%)	907071 (19·8)	93381 (20·1)	1000452 (19·8)
4 (50·0%–57·5%)	850499 (18·6)	98907 (21·3)	949406 (18·8)
5 (57·5%–100%)	752764 (16·4)	99942 (21·5)	852706 (16·9)
Vaccination status at baseline			
Unvaccinated	477967 (10·4)	66147 (14·2)	544114 (10·8)
Vaccinated with 1 dose	127922 (2·8)	20783 (4·5)	148705 (2·9)
Vaccinated with 2 doses	3852495 (84·0)	371939 (80·0)	4224434 (83·7)
Vaccinated with ≥3 doses	126077 (2·8)	5796 (1·2)	131873 (2·6)

^a^ Full list of demographic characteristics is provided in the S3 Table in S1 Appendix. Continuous variables were presented as mean (± Standard Deviation)· Categorical variables were presented as number of individuals and percentages. Standardized differences between the two groups were also presented with 95% confidence interval.

^b^ The sum of counts does not equal the column total because of individuals with missing information (≤1·0%) for this characteristic.

From November 22, 2021 to March 21, 2022, we observed 4,973 Omicron-associated hospitalizations, with a median follow-up time of 119 days and an incidence rate of 2·52 (95% CI, 2·45–2·59) per 10,000 person-months. We also observed 827 deaths following Omicron infection, with an incidence rate of 0·42 (95% CI, 0·39–0·45). A total of 5,568 cases with severe outcomes following Omicron infection was identified, with an incidence rate of 2·82 (95% CI, 2·75–2·90).

### Estimated protection against Omicron-associated hospitalization

We observed that in the overall cohort, infection-acquired immunity provides 67% (95% CI, 51–78) protection against Omicron-associated hospitalization among unvaccinated individuals. Similar protection was seen in the ≤64 year-old group (70%, 95% CI, 55–81) and the ≥65 year-old group (81%, 95% CI, 55–92).

**Fig 2:**
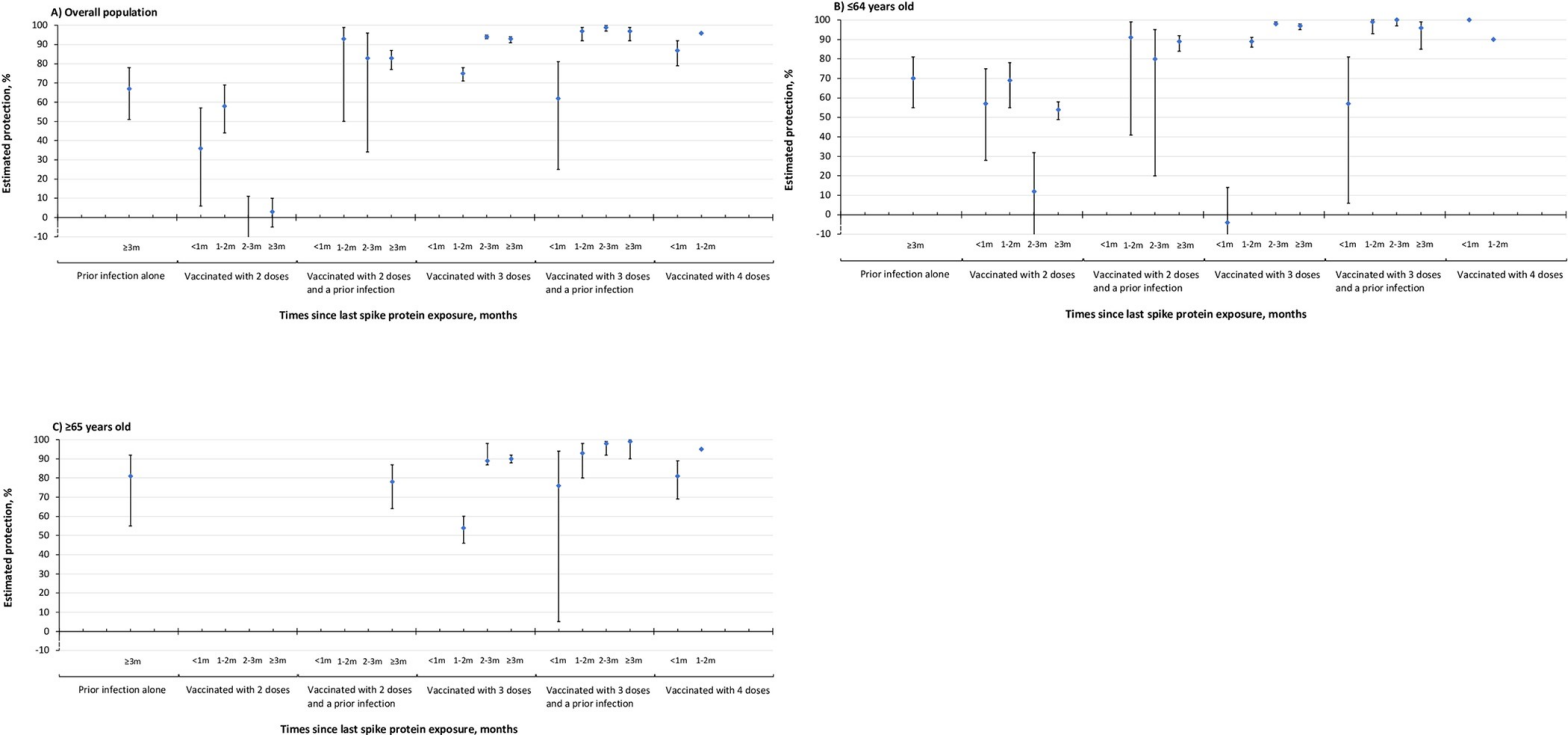
Estimated protection of a prior infection alone, COVID-19 vaccination, and hybrid immunity against hospitalization associated with Omicron infection in A) the overall cohort, B) individuals ≤64 years old, and C) individuals ≥65 years old. Individuals without a prior SARS-CoV-2 infection and unvaccinated serve as the reference group. Since a prior SARS-CoV-2 infection was determined by any positive PCR test at least 90 days before an Omicron infection, protection of a prior infection < 3 months since last infection was not applicable. In the overall cohort (A), estimated protection of 2 doses and a prior infection at <1 month and 3 doses at <1 month was not presented in the figure owing to insufficient cases and imprecision in the estimates with wide 95% Cls. In individual ≤64 years old (B), estimated protection of 2 doses and a prior infection at <1 month was not presented in the figure owing to insufficient cases and imprecision in the estimates with wide 95% Cls. In individuals ≥65 years old (C), estimated protection of 2 doses, 2 doses and a prior infection <3 months, and 3 doses at <1 month was not shown in the figure owing to insufficient cases and imprecision in the estimates with wide 95% Cls.

In the overall cohort (Fig 2A), among individuals with two vaccine doses, having a prior infection was associated with significantly higher protection against Omicron-associated hospitalization at 2–3 (83%, 95% CI, 34–96) and ≥3 months (83%, 95% CI, 77–87). Protection from a hybrid of three vaccine doses and a prior infection was 97% (95% CI, 92–99) at 1–2 months since the last antigenic exposure, significantly higher than three-dose vaccination alone (75%, 95% CI, 71–78). Three doses provided 94% (95% CI, 93–95) and 93% (95% CI, 91–94) protection at 2–3 months and ≥3 months after vaccination respectively, which were similar to the protection conferred by a combination of three doses and a prior infection over the same time periods (2–3 months: 99%, 95% CI, 97–100; ≥3 months: 97%, 95% CI, 92–99) and four doses in the first two months (<1 month: 87%, 95% CI, 79–92; 1–2 months: 96%, 95% CI, 92–98). We observed that among individuals with a third or a fourth dose, the protection was over 90% after the first month since the last antigenic exposure.

We observed similar trends in the ≤64 year-old population (Fig 2B). Three doses provided 89% (95% CI, 82–89) protection at 1–2 months post-vaccination, 98% (95% CI, 98–99) protection at 2–3 months, and 97% (95% CI, 98–99) protection at ≥3 months, which was comparable to the protection of a hybrid of three doses and a prior infection over the same time periods (1–2 months: 99%, 95% CI, 93–100; 2–3 months: 100%, 95% CI, 97–100; ≥3 months: 96%, 95% CI, 85–99) and four doses in the first two months post-vaccination (<1 month: 100%, 95% CI, 100–100; 1–2 months: 90%, 95% CI, 60–97). In the ≥65 year-old group (Fig 2C), protection of four vaccine doses increased to 95% (95% CI, 91–98) at 1–2 months post-vaccination, comparable to that of a hybrid of three doses and a prior infection ≥1 month since the last antigenic exposure but significantly higher than that of three doses alone over the follow-up period. In both age groups, we did not observe significant waning of protection in individuals with three doses, with or without a prior infection, after the first month since last antigenic exposure.

### Estimated protection against the composite severe outcome

Infection-acquired immunity offered a similar level of protection against Omicron-associated hospitalization and the composite severe outcome. Specifically, the protection of a prior infection alone against the latter was 68% (95% CI, 53–78) in the overall cohort, 72% (95% CI, 57–82) in the ≤64 year-old group, and 82% (95% CI, 57–93) in the ≥65 year-old group.

**Fig 3:**
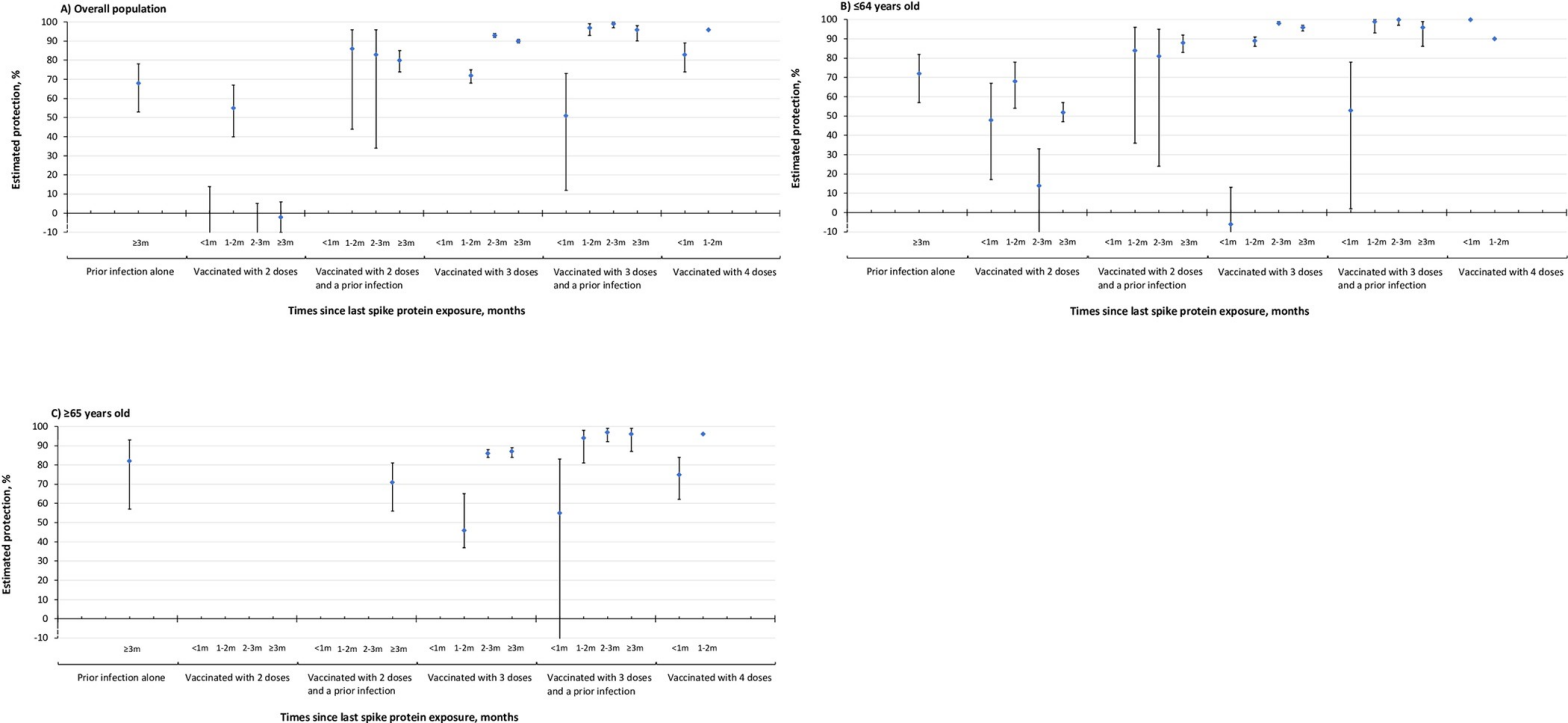
Estimated protection of a prior infection alone, COVID-19 vaccination, and hybrid immunity against composite severe outcome associated with Omicron infection in A) the overall cohort, B) individuals ≤64 years old, and C) individuals ≥65 years old. Individuals without a prior SARS-CoV-2 infection and unvaccinated serve as the reference group. Since a prior SARS-CoV-2 infection was determined by any positive PCR test at least 90 days before an Omicron infection, protection of a prior infection < 3 months since last infection was not applicable. In the overall cohort (A), estimated protection of 2 doses and a prior infection at <1 month, 3 doses at <1 month was not presented in the figure owing to insufficient cases and imprecision in the estimates with wide 95% Cls. In individual ≤64 years old (B), estimated protection of 2 doses and a prior infection at <1 month was not presented in the figure owing to insufficient cases and imprecision in the estimates with wide 95% Cls. In individuals ≥65 years old (C), estimated protection of 2 doses, 2 doses at < 3 months, and 3 doses at <1 month was not presented in the figure owing to insufficient cases and imprecision in the estimates with wide 95% Cls.

In the overall cohort (Fig 3A), compared to immunity acquired from two-dose vaccination only, a hybrid of two doses and a prior infection conferred higher level of immunity after the first month from the last antigenic exposure: 86% (95% CI, 44–96) at 1–2 months, 83% (95% CI, 34–96) at 2–3 months, and 80% (95% CI, 74–85) at ≥3 months. The protection offered by three doses remained consistent after two months post-vaccination, with 93% (95% CI, 92–94) and 90% (95% CI, 89–91) at 2–3 months and ≥3 months respectively. These values were comparable to the protection from a hybrid of three doses and a prior infection over the same periods (2–3 months: 99%, 95% CI, 97–100; ≥3 months: 96%, 95% CI, 90–98) and four doses in the first two months since vaccination (<1 month: 83%, 95% CI, 74–89; 1–2 months: 96%, 96% CI 93–98).

Among individuals ≤64 years old (Fig 3B), three vaccine doses provided 89% (95% CI, 86–91) protection at 1–2 months after vaccination, 98% (95% CI, 98–99) at 2–3 months, and 96% (95% CI, 94–97) at ≥3 months against the composite severe outcome. These results were comparable to those offered by a hybrid of three doses and a prior infection over the same time periods and by four doses within the first two months post-vaccination. In the ≥65 years old group (Fig 3C), protection of four doses increased to 96% (95% CI, 92–98) at 1–2 months post-vaccination, comparable to that of a hybrid of three doses and a prior infection after one month since the last antigenic exposure, but significantly higher than that of three-dose vaccination alone over the follow-up period. We did not observe significant waning of protection in individuals with three doses, regardless of having a prior infection, after the first month since last antigenic exposure.

## Discussion

In this cohort study with over five million individuals, we examined the protection of infection-acquired immunity and how it modifies immunity conferred by a third or fourth dose of COVID-19 vaccine in preventing severe outcomes associated with Omicron infection during a period of BA.1 and BA.2 predominance. Overall, three doses provided over 90% protection against Omicron-associated hospitalization and severe outcomes at 2–3 months and ≥3 months post-vaccination, which was similar to the protection conferred by three doses and a prior infection over the same time periods and four doses in the first two months. Among individuals ≥65 years old, protection of four doses increased in the second month post-vaccination and was significantly higher than that conferred by three vaccine doses. We also found that in both age groups, immunity acquired from infection and/or vaccination against Omicron-associated hospitalization and severe outcomes started increasing after the first month since the last vaccination or infection, and no significant waning of immunity acquired from boosters was observed during the follow-up period.

Our findings suggest that at least three antigenic exposures, achieved by either vaccination or a combination of infection and vaccination, can induce a high level of immunity to prevent Omicron-associated severe illness. This highlights that after giving booster doses to high-risk groups, infection-naïve individuals who completed two doses should be prioritized for a third vaccine dose over those previously infected. A third dose can provide additional protection even for those previously infected, and therefore should be rolled out among the overall population, regardless of their prior infection status, when sufficient vaccine supply is available. Our results are consistent with existing studies that reported 60%-90% protection acquired from three vaccine doses alone against Omicron-associated hospitalization and over 80% protection against hospitalization and death [7–9, 13, 15]. With over five million individuals in the cohort, our study adds significance to previous studies on hybrid immunity that reported over 90% protection of a hybrid of three doses and a prior infection against Omicron-associated hospitalization and death [7–9]. Although to our knowledge no studies to date have compared hybrid immunity to four doses, recent studies have reported high vaccine effectiveness of a fourth dose. For example, a study in Canada reported 86% protection of a fourth dose against Omicron-related severe outcomes [23]. Furthermore, our study showed that in individuals aged 65 years and older, four vaccine doses provided significantly higher protection than three doses only, highlighting the importance of recommending a fourth dose for this age group. These results also align with previous studies reporting improved effectiveness of a fourth dose compared with that of three doses only [23, 24]. These observations are supported by immunological evidence that increased number of antigenic exposures, rather than the type of exposure, strengthens the magnitude of the serum antibody neutralizing response and enhances cross-reactive T-cell responses, which are more resilient to mutations and likely to contribute to protection against severe disease [25–30].

Our results show that after the first month since last infection or vaccination, immunity acquired from three and four vaccine doses, as well as hybrid immunity, did not decline in the following three months. This suggests that individuals who receive boosters (a third or a fourth dose), regardless of having a prior infection, have durable immunity for at least three months after infection or vaccination. Our findings align with the current evidence that immunity acquired from a third or a fourth dose declines typically starting from at least four months post-vaccination [11, 18, 23, 31]. A systematic review of hybrid immunity also reported that protection of three vaccine doses and prior infection against Omicron-associated severe disease remained stable at over 95% within six months post-infection or vaccination [32]. As shown in the S5, S6 Tables in S1 Appendix, the lower protection of three vaccine doses and hybrid immunity against Omicron-associated hospitalization and severe outcomes that we observed in the first month following infection or vaccination suggests that it may take up to a month to fully develop immunity from vaccination. This observation is in contrast with most previous studies that showed the highest effectiveness of three and four doses in the first month after vaccination. Although evidence from immunological studies suggests that it takes about two to four weeks after the final dose of a COVID-19 vaccine for the body to develop maximum immunity, the exact timeline for developing immunity may vary depending on factors such as the type of vaccine, the person’s age, and their immune system [33, 34]. Finally, the large 95% CI, indicates that we had insufficient number of cases in the group with three doses at <1 month, which leads to imprecision in these estimates.

Finally, our findings also support previous studies that a prior infection offers a high level of cross-variant immunity against severe illness in the population. Our estimation was higher than a recently published cohort study, which reported only 28·5% protection against Omicron-related hospitalization [13], but lower than early studies reporting 80%-90% protection against Omicron-related hospitalization [7–9, 15]. This may be attributed to our cohort consisting of individuals who tested positive since the beginning of the pandemic. Therefore, immunity acquired from infection may have already been waning.

This study has the following limitations. First, our estimates may be biased by residual confounding that we were unable to account for in the models, including differences between vaccinated and unvaccinated groups and previously infected and infection-naïve groups in terms of mobility and contact levels, compliance with public health and social measures, and susceptibility to infection [35]. Second, with over 30% of our cohort older than 50 years of age, our findings may not be generalizable to settings with a much younger population. However, we stratified our analysis by age, which provides insights into how age might influence vaccination-induced and hybrid immunity against the Omicron variant. Third, we may have missed some SARS-CoV-2 infections both pre-Omicron and after Omicron emergence because of asymptomatic cases. Fourth, changes in the testing policies, such as the rise in rapid antigen testing that our data did not account for, and the reduced availability of RT-PCR testing, could have influenced our findings. Although the direction of the effect due to these changes on our results is uncertain, a previous study showed that the effect of the vaccines would be underestimated if vaccinated individuals were more likely to be tested than unvaccinated individuals [36]. Similarly, exposure misclassification may occur if the timing or the number of doses of vaccination, or the history of previous infection, were not captured in the databases or incorrectly recorded. Moreover, our analysis could potentially be biased due to misclassification of outcomes, which may result from reporting delays, cases not being linked correctly, and the databases failing to capture all severe COVID-19 cases. The extent and direction of this bias depends on whether the differences in data completeness and reporting delays are differential between the vaccinated and unvaccinated groups who have tested positive. For instance, if unvaccinated individuals experience more severe illnesses but their admissions are not recorded promptly, our vaccine effectiveness could be underestimated. Fifth, owing to our short follow-up window and a small number of individuals who received a fourth dose, the majority of the population vaccinated with a third or a fourth dose was over 60 years old (S4 Table in S1 Appendix) and we were not able to observe protection of three and four doses alone and hybrid immunity beyond three months. As many countries have expanded the eligibility for a fourth dose to the general population, observational studies that assess the long-term effect of third and fourth dose are needed to provide real-world evidence and inform vaccination policies. Finally, we acknowledge that vaccine effectiveness may vary between vaccine types. However, our analysis was not stratified by vaccine type owing to insufficient data on this aspect. Consequently, there is a need for further studies that can examine the nuances of protection afforded by each vaccine type against various COVID-19 strains.

## Conclusion

Our study is one of the largest to examine and compare the protection of prior infection, a third and a fourth COVID-19 vaccine dose, and hybrid immunity against Omicron-associated severe illness. We found that at least three antigenic exposures, achieved by vaccination only or a hybrid of vaccination and infection, can confer high protection against Omicron-associated hospitalization and death in both younger and older populations for at least three months. Based on these findings, we recommend a third dose for the overall population, regardless of prior infection status, and a fourth dose for the elderly to maintain high level of immunity and substantially reduce risk of severe illness at individual level.

## Supporting information

S1 Appendix (DOCX)
