# Supplementary material for: Protection of prior SARS-CoV-2 infection, COVID-19 boosters, and hybrid immunity against Omicron severe illness: A population-based cohort study of five million residents in Canada
Source: PLoS One. 2024 Feb 23;19(2):e0299304. doi: 10.1371/journal.pone.0299304 (PMC10889649; doi:10.1371/journal.pone.0299304)
Supplement: S1 Appendix — (DOCX) [file pone.0299304.s001.docx]

**Supplementary material**

**Table of content**

p2. Section S1. Details about variant ascertainment

p3. Table S1. Definitions of covariates

p7. Table S2. Definitions of comorbidities

p15. Section S2. Strengthening the Reporting of Observational studies in Epidemiology (STROBE) checklist for cohort studies

p18. Table S3. Complete list of demographic characteristics and vaccination status at baseline

p20. Table S4. Demographic characteristics of participants vaccinated with three and four doses at baseline

p22. Table S5. Estimated protection of a past SARS-CoV-2 infection, COVID-19 vaccines, and a hybrid immunity against Omicron-associated hospitalization, stratified by age and time since last infection/vaccination

p24. Table S6. Estimated protection of a past SARS-CoV-2 infection, COVID-19 vaccines, and a hybrid immunity against Omicron-associated composite severe outcome, stratified by age and time since last infection/vaccination

p26. References

**Section S1. Details about variant ascertainment**

Infection with the Omicron variant was identified by whole genome sequencing (WGS) as the B.1.1.529 lineage or having S-gene target failure (SGTF) detected before December 21, 2021. From December 21, 2021 onwards, any positive SARS-CoV-2 infection cases were classified as Omicron cases (unless confirmed as other variants by WGS or SGTF screening), based on projections that more than 80% of cases were likely to be Omicron by this date.^1^ SGTF had been used to identify Omicron in Ontario starting on December 6, 2021. Between December 6 and 24, 2021, all specimens with a positive PCR result (and a cycle threshold [Ct] value ≤35) were sent for SGTF testing using the Thermofisher TaqpathTM COVID-19 PCR.^1^ Prior to December 20, SGTF-positive specimens with Ct values ≤30 also underwent WGS. SGTF is a reliable proxy for Omicron identification, with 98·9% sensitivity and 99·9% specificity during December 2021 and January 2022 in Ontario.^1^

**Table S1. Definitions of covariates**

| **Variables** | **Definition** |
| --- | --- |
| Age | Age was determined from the Registered Persons Database. |
| Sex | Sex was determined from the Registered Persons Database. |
| Rural residence | Residence was distinguished between rural and urban. Information about rural areas was matched to individuals using postal code. |
| Long-term care (LTC) resident | Contact with long-term cares (LTC), either a resident or a worker, 90 days prior to the testing date was included as risk factor because high mortality in LTC facilities was reported in several countries, including Canada. |
| Dissemination Area (DA) | A dissemination area (DA) is the smallest standard geographic area for which all census data are disseminated. A DA generally comprises approximately 400-700 people, but in densely populated cities may contain several thousand people. DAs cover all the territory of Canada.^2^  We assigned subjects to a DA using postal code, as recorded in OLIS. For those who did not have a valid postal code recorded or were not tested for COVID-19, we used the individual’s postal code as listed in the Registered Persons Database. |
| Public health unit region | Taken from Public Health Unit (PHU) information using postal code of residence as recorded in the Registered Persons Database and Statistics  Canada Postal Code Conversion File Plus (version 7B). Regions were defined as follows:  Central East: PHU 35 (Haliburton, Kawartha, Pine Ridge District Health Unit), 55 (Peterborough County—City Health Unit), 60 (Simcoe Muskoka District Health Unit)  Central West: PHU 27 (Brant County Health Unit), 34 (Haldimand-Norfolk Health Unit), 36 (Halton Regional Health Unit), 37 (City of Hamilton Health Unit), 46 (Niagara Regional Area Health Unit), 65 (Waterloo Health Unit), 66 (Wellington-Dufferin-Guelph Health Unit)  Durham: PHU 30 (Durham Regional Health Unit)  Eastern: PHU 38 (Hastings and Prince Edward Counties Health Unit), 41 (Kingston, Frontenac and Lennox and Addington Health Unit), 43 (Leeds, Grenville and Lanark District Health Unit), 57 (Renfrew County and District Health Unit), 58 (The Eastern Ontario Health Unit)  North: PHU 26 (The District of Algoma Health Unit), 47 (North Bay Parry Sound District Health Unit), 49 (Northwestern Health Unit), 56 (Porcupine Health Unit), 61 (Sudbury and District Health Unit), 62 (Thunder Bay District Health Unit), 63 (Timiskaming Health Unit)  Ottawa: PHU 51 (City of Ottawa Health Unit)  Peel: PHU 53 (Peel Regional Health Unit)  South West: PHU 31 (Elgin-St. Thomas), 33 (Grey Bruce Health Unit), 39 (Huron County Health Unit), 40 (Chatham-Kent Health Unit), 42 (Lambton Health Unit), 44 (Middlesex-London Health Unit), 52 (Oxford), 54 (Perth District Health Unit), 68 (Windsor-Essex County Health Unit), 75 (Southwestern Health Unit)  Toronto: PHU 95 (City of Toronto Health Unit)  York: PHU 70 (York Regional Health Unit) |
| Household income quintile | Calculated at the DA level using 2016 Census data by multiplying the median income (before-tax) by the number of households and dividing by the sum of single-person equivalent to obtain income per single person equivalent. For DAs where median income was unavailable, neighboring DAs were used to estimate income per single person equivalent. DA-based income quintiles were constructed separately for  each census metropolitan area or census agglomeration (one or more adjacent municipalities integrated via commuting flows). DAs within each such area were ranked from the lowest average income per single-person equivalent to the highest, and DAs were assigned to five groups, such that each group contained approximately one-fifth the total in-scope population of each area. |
| Essential worker quintile | Calculated at the DA level, using 2016 Census data. For each DA, we calculated the number of individuals ≥15 years old that were working in one of the following Census-defined work categories: Sales and service occupations; trades, transport and equipment operators and related occupations; natural resources, agriculture and related production occupations; and occupations in manufacturing and utilities.  DAs across the province were then ranked by these percentages into quintiles, with the lowest 1/5 of DAs comprising the first quintile, and so on.^3^ |
| Persons per dwelling quintile | Average number of persons in private households, calculated at the DA level using the 2016 Census data. DAs across the province were ranked by average number of persons per household into 5 categories (quintiles), such that each group contained approximately one-fifth of the DAs.^3^ |
| Limited educational attainment quintile | Calculated at the DA level using 2016 Census data.^4^ This variable was computed by identifying the number of adults aged 25-64 reporting having “no certificate, diploma, or degree” and dividing this value by the total number of individuals aged 25-64 having answered questions about their highest certificate, diploma or degree. This yielded a percentage of individuals in each DA considered to have no certificate, diploma, or degree. DAs across the province were then ranked by these percentages into quintiles, with the lowest 1/5 of DAs comprising the first quintile, and so on. |
| Uncoupled quintile | Calculated at the DA level using 2016 Census data.^5^ “Uncoupled” individuals are individuals who, in the 2016 Census, reported having the following relationship statuses (wording from 2016 Census): never married (persons who have never legally married and are not living with a person as a couple); separated (persons who are married but who are no longer living with their spouse [for reasons other than, for example, illness, work or school], have not obtained a divorce and are not living with a person as a couple; divorced (persons who have obtained a legal divorce, have not remarried and are not living with a person as a couple); and widowed (persons who have lost their married spouse through death, have not remarried and are not living with a person as a couple).  The number of individuals fulfilling this description in a given DA was divided by the total number of individuals with marital status in the DA, yielding a percentage figure for each DA. DAs across the province were then ranked by these percentages into quintiles, with the lowest 1/5 of DAs comprising the first quintile, and so on. |
| Self-identified visible minority quintile | Calculated at the DA level, using 2016 Census data. An individual was marked as “self-identify as a visible minority” if they reported being one or more of the following (wording from the 2016 Census): “South Asian (e.g., East Indian, Pakistani, Sri Lankan, etc.), Chinese, Black, Filipino, Latin American, Arab, Southeast Asian (e.g., Vietnamese, Cambodian, Laotian, Thai, etc.), West Asian (e.g., Iranian, Afghan, etc.), Korean, Japanese, or Other—specify”.  DAs across the province were then ranked by these percentages into quintiles, with the lowest 1/5 of DAs comprising the first quintile, and so on.^3^ |
| Recent immigration category | Calculated at the DA level, using 2016 Census data.^6^ This value was obtained by identifying the number of individuals in a DA that reported having immigrated in the past 5 years, and dividing this number by the number of individuals in the DA who reported their immigration status over the past five years (i.e., were immigrants or non-immigrants, regardless of the time period of immigration).  DAs across the province were then ranked by percentage of recent percentages into three groups with cut-offs at the 60th and 80th percentiles, due to a zero-inflated distribution of DAs. |

**Table S2. Definitions of comorbidities**

| **Comorbidity** | **Case definition** | **Codes** | **Validation** |
| --- | --- | --- | --- |
| Asthma | At least one hospitalization admission with a diagnosis of asthma or two physician claims of asthma diagnosis in a two-year period. An ICES-specific asthma database was used to identify patients with asthma. | ICD-9/OHIPDX: 493  ICD-10: J45, J46 | Sensitivity = 81%  Specificity = 81%^7^ |
| COPD | At least 1 physician billings within 2 years or one hospitalization with a COPD diagnosis code prior to index date. An ICES-specific COPD database was used to identify patients with COPD. | ICD-9/OHIPDX: 491, 492, 496  ICD-10: J41, J42, J43, J44 | Sensitivity = 85%  Specificity = 79%^8^ |
| Dementia | Over 40 years old, at least one hospitalization admission or one prescription in the Ontario Drug Benefit program for cholinesterase inhibitors or over three physician claims at least 30 days apart in a one-year period | ICD-9: 46.1, 290.0, 290.1, 290.2, 290.3, 290.4, 294.x, 331.0,  331.1, 331.5, 331.82  ICD-10: F00.x, F01.x, F02.x,  F03.x, G30.x  OHIP: 290, 331 | Sensitivity = 79%  Specificity = 99%^9^ |
| HIV | At least three physician claims for HIV diagnosis in a three-year period. An ICES-specific HIV database was used to identify patients with HIV. | OHIPDX: 042, 043, 044 | Sensitivity = 96%  Specificity = 99%^10^ |
| Hypertension | At least one hospital admission with a diagnosis of hypertension or two physician claims for hypertension within two years was included. Cases of gestational hypertension were excluded. An ICES-specific hypertension database was used to identify patients with hypertension. | ICD-9/OHIPDX: 401x, 402x, 403x, 404x, 405x  ICD-10: I10, I11, I12, I13, I15 | Sensitivity = 73%  Specificity = 95%^11^ |
| Diabetes | Age <= 18:  At least four physician claims with a diabetes diagnosis code in a two-year period OR at least one physician claims with a diabetes fee code.  Age 19+:  At least one hospital admission with a diagnosis of diabetes or two physician claims for diabetes or one prescription claim for diabetes medications within 1 year. Cases of gestational diabetes were excluded.  An ICES-specific diabetes database was used to identify patients with diabetes. | ICD-9/OHIPDX: 250  ICD-10: E10, E11, E13, E14  OHIP Feecode: Q040, K029, K0303, K045, K046  ODB DINS: INSULIN or ORAL ANTI-GLYCEMICS | Age <=18:  Sensitivity: 83%  Specificity: 99%  Age 19+  Sensitivity = 90% Specificity = 98%^12^ |
| Chronic kidney diseases | At least one hospital admission with a diagnosis of chronic kidney diseases, or one physician claim, or emergency department visit for a diagnosis of chronic kidney diseases within the last five years.  Or receiving at least one dialysis billing code in each of the three months prior to Jan 15, 2020. | Diagnosis code:  ICD-9: 4030; 4031; 4039; 4040; 4041; 4049; 585; 586; 5888; 5889; 2504  ICD-10: E102, E112, E132, E142, I12, I13, N08, N18, N19  OHIP dx: 403, 585  Dialysis code:  CCP: 5195, 6698  CCI: 1PZ21HQBS, 1PZ21HQBR, 1PZ21HPD4  OHIP: R849, G323, G325, G326, G860, G862, G863, G865, G866, G082, G083, G085, G090, G091, G092, G093, G094, G095, G096, G294, G295, G330, G331, G332, G861, G864, G333, H540, H740 | Sensitivity = 33%  Specificity > 94%^13^ |
| Cancer | A history of cancer based on at least one diagnosis in the Ontario Cancer Registry (OCR) prior to January 15, 2020. The OCR contains information on all malignant cancers (except for non-melanoma skin cancers) diagnosed in Ontario. It is a passive registry that is comprised of information from 4 data major sources: cancer-related hospital and surgical records (from CIHI DAD and NACRS data); pathology reports; records from Ontario’s Regional Cancer Centers; and death certificates with a mention of cancer. |  | Not validated. |
| Congestive heart failure | At least one hospital admission with a congestive heart failure diagnosis, one physician claim/emergency department visit with a congestive heart failure diagnosis followed within one year by a second record from either source or one hospital admission. | ICD-9/OHIPDX: 428  ICD-10: I500, I501, I509 | Sensitivity = 85%  Specificity = 97%^14^ |
| Cardiac ischemic disease | At least one hospitalization admission with a diagnosis of cardiac ischemic diseases (angina, chronic ischemic heart disease, or myocardial infarction) in the past five years.  Or history of receiving the cardiac procedures of coronary artery bypass grafting or percutaneous coronary interventions in the past 20 years.^15^ | Diagnosis code  ICD-10: I20, I25, I21, I22  Procedure code:  CCI: 1IJ76, 1IJ50, 1IJ5, 1IJ57  CCP: 481, 4802, 4803 | Not validated |
| Transient ischemic stroke | At least one hospitalization admission or one emergency department visit with a diagnosis of transient ischemic stroke.^16^ | Diagnosis code  ICD-9: 435, 3623  ICD-10: G450, G451, G452, G453, G458, G459, H340 | Not validated. |
| Acute ischemic stroke | At least one hospitalization admission or emergency department visit with a main acute ischemic stroke diagnosis. | Diagnosis code  ICD-9: 434, 436  ICD-10: I63 (excluding I63.6), I64, H34.1 | Not validated. |
| Hemorrhagic stroke | At least one hospitalization admission or emergency department visit with a diagnosis of hemorrhagic stroke. | Diagnosis code:  ICD-9: 430, 431  ICD-10: I60, I61 | Not validated. |
| Rheumatoid arthritis | At least one hospitalization admission with a diagnosis of rheumatoid arthritis, or at least three diagnostic codes of rheumatoid arthritis over two years with at least one provided by a musculoskeletal specialist (rheumatology, orthopedic surgery, or internal medicine). | ICD-9/OHIPDX: 714  ICD-10: M05, M06 | Sensitivity = 78%  Specificity = 100%^17^ |
| Inflammatory bowel disease | Age <18:  At least one OHIP procedure code for sigmoidoscopy/colonoscopy and at least two hospitalizations or at least four physician billings in OHIP or emergency department visits in a three year period OR at least three hospitalizations or at least seven physician billings in OHIP or emergency department visits in a three year period (if no OHIP procedure)  Age 18-64:  Two years of OHIP eligibility and at least five hospital, emergency department, and/or physician billing records for Crohn’s disease or ulcerative colitis in a four year period OR at least three hospital, emergency department, and/or physician billing records for Crohn’s disease or ulcerative colitis in a four year period (no 2-year OHIP eligibility)  Age 65+:  Two years of OHIP eligibility and at least five hospital, emergency department, and/or physician billing records for Crohn’s disease or ulcerative colitis in a four year period and at least one ODB claim for IBD medication OR at least three hospital, emergency department, and/or physician billing records for Crohn’s disease or ulcerative colitis in a four year period and at least 1 ODB claim for IBD medication (no 2-year OHIP eligibility) | ICD-9/OHIPDX: 555, 556  ICD-10: K50. K51  OHIP procedure Feecode: Z535, Z555, Z580, E740, E741, E7417, E705 | Children/youth (<18):  Sensitivity = 91%  Specificity = 99%^18^  Adults (18-64):  Sensitivity = 77%  Specificity = 96%^19^  Older adults (65+):  Sensitivity = 59%  Specificity = 99% |
| Liver disease | Patients who have at least one hospitalization admission or two outpatient visits (physician or emergency department visits) with a cirrhosis diagnosis OR patients who have at least one outpatient visit and one hospitalization admission one procedure for decompensated cirrhosis. | Diagnosis code for Cirrhosis  OHIP dx code: 571  ICD-9: 456.1, 571.2, 571.5  ICD-10: I85.9, I98.2, K70.3, K71.7, K74.6  Diagnostic code for Decompensated Cirrhosis  ICD-9: 456.0, 456.2, 572.2, 572.3, 572.4, 782.4, 789.5  ICD-10: I85.0, I86.4, I98.20, I98.3, K721, K729, K76.6, K76.7, R17, R18  Procedure codes:  CCI: 1. NA.13.BA-FA, 1.NA.13.BA-X7, 1.NA.13.BA-BD, 1.KQ.76GP-NR, 1.OT.53.HA  CCP: 1006, 6691  OHIP: J057, Z591 | Sensitivity = 88 - 99%  Specificity = 89 - 98%^20^ |
| Severe mental illness | Individuals admitted to an acute care or psychiatric facility in the 2 years prior to index date for anxiety, deliberate self-harm, mood disorders (bipolar, depression), obsessive compulsive and related disorders, personality disorders, schizophrenia spectrum and other psychotic disorders, substance-related and addictive disorders, and trauma or stressor-related disorders.^16^ | Substance-Related and Addictive Disorders  ICD-9: 291.x (all 291 codes), 292.x (all 292 codes), 303.x (all 303 codes), 304.x (all 304 codes), 305.x. Provisional=16 can be split as sub-groups:  291.x, 303.x, 3050 = ALCOHOL; 3040, 3047, 3055 = OPIOIDS; 292.x, 304 [excl. 3040, 3047], 305 [excl.3050, 3055] = OTHERDRUGS  ICD-10: F10.x-F19.x, Z72.0. Provisional=16  can be split as sub-groups:  F10 = ALCOHOL  F11 = OPIOIDS  F12, F13, F14, F15, F16, F18, F19 = OTHERDRUGS  F17, Z720 = OTHER  Schizophrenia Spectrum and Other Psychotic Disorders  ICD-9: 293.81/82, 295.x (all 295 codes), 297.x (all 297 codes), 298.x (all 298 codes). Provisional=2  ICD-10: F20.81, F20.9, F22, F23, F25, F06.0/1/2, F28, F29. Provisional=2  Mood disorders  ICD-9: 293.83, 296.x (all 296 codes), 300.4x, 301.13, 311.x, 625.4. Provisional=3, 4 Can be split as follows: Bipolar [296.0x, 296.4x, 296.5x, 296.6x, 296.7x, 296.8x, 301.13. provisional=3], Depressive [296.2x, 296.3x, 296.9x, 300.4x, 311.x, 625.4x. provisional=4], Other mood [293.83]  ICD-10: F06.3, F31, F32, F33, F34. Provisional = 3, 4  Can be split as follows: Bipolar [F31, F34.0, F06.33, F06.34], Depressive [F32, F33, F34.81, F34.1, F06.31, F06.32]  Anxiety disorders  ICD-9: 293.84, 300, 300.0x, 300.2x, 309.21, 313.23. Provisional=5  ICD-10: F06.4, F40.0x, F40.1x. F40.2x, F41.0x/1x, F41.8x/9x, F93.0, F94.0. Provisional=5  Trauma/stressor-related disorders  ICD-9: 308.3x, 309, 309.0x, 309.24, 309.28, 309.3x, 309.4x, 309.81, 309.89, 309.9x, 313.89. Provisional=7  ICD-10: F43.0, F43.1. F43.2, F43.8/9, F94.1/2. Provisional=7  OCD & related disorders  ICD-9: 300.3x, 300.7x, 312.39, 698.4x. Provisional=6  ICD-10: F06.8, F42.2x, F42.3, F42.4, F42.8, F42.9, F45.2, F63.3. Provisional=6  Personality disorders  ICD-9: 301, 301.0x, 301.2x, 301.4x, 301.5x, 301.6x, 301.7x, 301.81-3, 301.89, 301.9x 310.1. Provisional=18  ICD-10: F07.x (all F07 codes), F21, F60. Provisional=18 | Not validated. |
| Solid organ transplant | Individuals who had history of solid organ transplant.^16^ | History of solid organ transplant, CCP/CCI codes:  1PC85 (Kidney) 1HZ85 (Heart) 1GR85, 1GT85 (Lung) 1HY85 (Heart & lung) 1OA85 (Liver) 1OK85 (Pancreas) 1OB85 (Spleen) | Not validated. |

**Section S2. Strengthening the Reporting of Observational studies in Epidemiology (STROBE) checklist for cohort studies**

|  | Item No | Recommendation | Pages |
| --- | --- | --- | --- |
| **Title and abstract** | 1 | (*a*) Indicate the study’s design with a commonly used term in the title or the abstract | Page 1 |
|  |  | (*b*) Provide in the abstract an informative and balanced summary of what was done and what was found | Page 2 |
| Introduction | | |  |
| Background/rationale | 2 | Explain the scientific background and rationale for the investigation being reported | Page 3 |
| Objectives | 3 | State specific objectives, including any prespecified hypotheses | Page 3 |
| Methods | | |  |
| Study design | 4 | Present key elements of study design early in the paper | Page 3 |
| Setting | 5 | Describe the setting, locations, and relevant dates, including periods of recruitment, exposure, follow-up, and data collection | Page 3 |
| Participants | 6 | (*a*) Give the eligibility criteria, and the sources and methods of selection of participants. Describe methods of follow-up | Page 3 |
|  |  | (*b*) For matched studies, give matching criteria and number of exposed and unexposed | Not applicable |
| Variables | 7 | Clearly define all outcomes, exposures, predictors, potential confounders, and effect modifiers. Give diagnostic criteria, if applicable | Page 4-5 |
| Data sources/ measurement | 8* | For each variable of interest, give sources of data and details of methods of assessment (measurement). Describe comparability of assessment methods if there is more than one group | Page 3; Table S1, Table S2 |
| Bias | 9 | Describe any efforts to address potential sources of bias | Section S3 |
| Study size | 10 | Explain how the study size was arrived at | Not applicable |
| Quantitative variables | 11 | Explain how quantitative variables were handled in the analyses. If applicable, describe which groupings were chosen and why | Page 4 |
| Statistical methods | 12 | (*a*) Describe all statistical methods, including those used to control for confounding | Page 5 |
|  |  | (*b*) Describe any methods used to examine subgroups and interactions | Page 5 |
|  |  | (*c*) Explain how missing data were addressed | Page 5 |
|  |  | (*d*) If applicable, explain how loss to follow-up was addressed | Not applicable |
|  |  | (*e*) Describe any sensitivity analyses | None |
| Results | | |  |
| Participants | 13* | (a) Report numbers of individuals at each stage of study—eg numbers potentially eligible, examined for eligibility, confirmed eligible, included in the study, completing follow-up, and analysed | Page 5; Figure 1 |
|  |  | (b) Give reasons for non-participation at each stage | Figure 1 |
|  |  | (c) Consider use of a flow diagram | Figure 1 |
| Descriptive data | 14* | (a) Give characteristics of study participants (eg demographic, clinical, social) and information on exposures and potential confounders | Page 5; Table 1 |
|  |  | (b) Indicate number of participants with missing data for each variable of interest | Table 1 |
|  |  | (c) Summarise follow-up time (eg, average and total amount) | Page 5 |
| Outcome data | 15* | Report numbers of outcome events or summary measures over time | Page 5 |
| Main results | 16 | (*a*) Give unadjusted estimates and, if applicable, confounder-adjusted estimates and their precision (eg, 95% confidence interval). Make clear which confounders were adjusted for and why they were included | Pages 5-7; Table S5, S6, S7 |
|  |  | (*b*) Report category boundaries when continuous variables were categorized | Pages 5-7 |
|  |  | (*c*) If relevant, consider translating estimates of relative risk into absolute risk for a meaningful time period | Not applicable |
| Other analyses | 17 | Report other analyses done—eg analyses of subgroups and interactions, and sensitivity analyses | Pages 5-7; Table S5, S6, S7 |
| Discussion | | |  |
| Key results | 18 | Summarise key results with reference to study objectives | Page 7 |
| Limitations | 19 | Discuss limitations of the study, taking into account sources of potential bias or imprecision. Discuss both direction and magnitude of any potential bias | Page 8 |
| Interpretation | 20 | Give a cautious overall interpretation of results considering objectives, limitations, multiplicity of analyses, results from similar studies, and other relevant evidence | Page 8 |
| Generalisability | 21 | Discuss the generalisability (external validity) of the study results | Page 8 |
| Other information | | |  |
| Funding | 22 | Give the source of funding and the role of the funders for the present study and, if applicable, for the original study on which the present article is based | Page 9 |

*Give information separately for exposed and unexposed groups.

**Note:** An Explanation and Elaboration article discusses each checklist item and gives methodological background and published examples of transparent reporting. The STROBE checklist is best used in conjunction with this article (freely available on the Web sites of PLoS Medicine at http://www.plosmedicine.org/, Annals of Internal Medicine at http://www.annals.org/, and Epidemiology at http://www.epidem.com/). Information on the STROBE Initiative is available at http://www.strobe-statement.org.

**Table S3. Complete list of demographic characteristics and vaccination status at baseline^a^**

| **Characteristics** | **Prior infection** | | **Total individuals**  **(N= 5,049,126)** |
| --- | --- | --- | --- |
|  | **No**  **(n= 4,584,461)** | **Yes**  **(n= 464,665)** |  |
| Age group, N (%) (years) |  |  |  |
| 12-19 | 477508 (10.4) | 54447 (11.7) | 531955 (10.5) |
| 20-29 | 812737 (17.7) | 100257 (21.6) | 912994 (18.1) |
| 30-39 | 825057 (18.0) | 87076 (18.7) | 912133 (18.1) |
| 40-49 | 703236 (15.3) | 79538 (17.1) | 782774 (15.5) |
| 50-59 | 729788 (15.9) | 76263 (16.4) | 806051 (16.0) |
| 60-69 | 549152 (12.0) | 42133 (9.1) | 591285 (11.7) |
| 70-79 | 308033 (6.7) | 16927 (3.6) | 324960 (6.4) |
| ≥80 | 178950 (3.9) | 8024 (1.7) | 186974 (3.7) |
| Sex, N (%) |  |  |  |
| Male | 2103205 (45.9) | 229780 (49.5) | 2332985 (46.2) |
| Female | 2481256 (54.1) | 234885 (50.5) | 2716141 (53.8) |
| Rural residence, N (%) |  |  |  |
| No | 4112660 (89.7) | 443062 (95.4) | 4555722 (90.2) |
| Yes | 460589 (10.0) | 20453 (4.4) | 481042 (9.5) |
| Neighborhood income quintile ^b^ |  |  |  |
| 1 (lowest) | 845098 (18.4) | 111649 (24.0) | 956747 (18.9) |
| 2 | 873416 (19.1) | 98819 (21.3) | 972235 (19.3) |
| 3 | 916082 (20.0) | 99546 (21.4) | 1015628 (20.1) |
| 4 | 945846 (20.6) | 84491 (18.2) | 1030337 (20.4) |
| 5 (highest) | 991010 (21.6) | 68812 (14.8) | 1059822 (21.0) |
| Public Health Unit ^b^ |  |  |  |
| Central East | 322953 (7.0) | 15083 (3.2) | 338036 (6.7) |
| Central West | 888654 (19.4) | 78778 (17.0) | 967432 (19.2) |
| Durham | 213523 (4.7) | 22232 (4.8) | 235755 (4.7) |
| Eastern | 291463 (6.4) | 9620 (2.1) | 301083 (6.0) |
| North | 281138 (6.1) | 9662 (2.1) | 290800 (5.8) |
| Ottawa | 312464 (6.8) | 22085 (4.8) | 334549 (6.6) |
| Peel | 446996 (9.8) | 87815 (18.9) | 534811 (10.6) |
| South West | 534254 (11.7) | 40618 (8.7) | 574872 (11.4) |
| Toronto | 907946 (19.8) | 130778 (28.1) | 1038724 (20.6) |
| York | 373858 (8.2) | 46844 (10.1) | 420702 (8.3) |
| Number of comorbidities, N (%) |  |  |  |
| 0 | 2561392 (55.9) | 282177 (60.7) | 2843569 (56.3) |
| 1 | 1242167 (27.1) | 120313 (25.9) | 1362480 (27.0) |
| 2 | 448104 (9.8) | 39365 (8.5) | 487469 (9.7) |
| ≥3 | 332798 (7.3) | 22810 (4.9) | 355608 (7.0) |
| Essential workers quintile ^b^ |  |  |  |
| 1 (0%–32.5%) | 1019286 (22.2) | 72616 (15.6) | 1091902 (21.6) |
| 2 (32.5%–42.3%) | 1032954 (22.5) | 97692 (21.0) | 1130646 (22.4) |
| 3 (42.3%–49.8%) | 907071 (19.8) | 93381 (20.1) | 1000452 (19.8) |
| 4 (50.0%–57.5%) | 850499 (18.6) | 98907 (21.3) | 949406 (18.8) |
| 5 (57.5%–100%) | 752764 (16.4) | 99942 (21.5) | 852706 (16.9) |
| Persons per dwelling quintile ^b^ |  |  |  |
| 1 (0-2.1) | 861544 (18.8) | 61048 (13.1) | 922592 (18.3) |
| 2 (2.2-2.4) | 802289 (17.5) | 57757 (12.4) | 860046 (17.0) |
| 3 (2.5-2.6) | 634560 (13.8) | 49398 (10.6) | 683958 (13.5) |
| 4 (2.7-3.0) | 1095203 (23.9) | 110725 (23.8) | 1205928 (23.9) |
| 5 (3.1-5.7) | 1166624 (25.4) | 183407 (39.5) | 1350031 (26.7) |
| Uncoupled quintile ^b^ |  |  |  |
| 1 (11.2 - 33.7%) | 1033038 (22.5) | 78124 (16.8) | 1111162 (22.0) |
| 2 (33.7 - 38.4%) | 895435 (19.5) | 85426 (18.4) | 980861 (19.4) |
| 3 (38.5 - 43.6%) | 847562 (18.5) | 93313 (20.1) | 940875 (18.6) |
| 4 (43.6 - 51.0%) | 864952 (18.9) | 100400 (21.6) | 965352 (19.1) |
| 5 (51.0 - 94.6%) | 919233 (20.1) | 105072 (22.6) | 1024305 (20.3) |
| Limited educational attainment quintile ^b^ |  |  |  |
| 1 (0.0-4.1%) | 1009334 (22.0) | 73920 (15.9) | 1083254 (21.5) |
| 2 (4.1 - 7.5%) | 1000482 (21.8) | 87888 (18.9) | 1088370 (21.6) |
| 3 (7.5 - 11.4%) | 954118 (20.8) | 101308 (21.8) | 1055426 (20.9) |
| 4 (11.4 - 17.1%) | 861988 (18.8) | 99297 (21.4) | 961285 (19.0) |
| 5 (17.1 - 94.3%) | 736669 (16.1) | 100125 (21.5) | 836794 (16.6) |
| Self-identified visible minority quintile ^b^ |  |  |  |
| 1 (0.0 - 2.2%) | 722048 (15.7) | 33764 (7.3) | 755812 (15.0) |
| 2 (2.2 - 7.5%) | 794670 (17.3) | 44484 (9.6) | 839154 (16.6) |
| 3 (7.5 - 18.7%) | 866246 (18.9) | 64350 (13.8) | 930596 (18.4) |
| 4 (18.7 - 43.5%) | 1023135 (22.3) | 106865 (23.0) | 1130000 (22.4) |
| 5 (43.5 - 102%) | 1156554 (25.2) | 213080 (45.9) | 1369634 (27.1) |
| Recent immigration quintile ^b^ |  |  |  |
| 1-3 (0.0 - 2.1%) | 2407535 (52.5) | 163925 (35.3) | 2571460 (50.9) |
| 4 (2.1 - 4.7%) | 952230 (20.8) | 104568 (22.5) | 1056798 (20.9) |
| 5 (4.7 - 41.2%) | 1176952 (25.7) | 192446 (41.4) | 1369398 (27.1) |
| Number of SARS-CoV-2 PCR tests | 2.8 (4.9) | 2.8 (4.4) | 2.8 (4.8) |
| Vaccination status at baseline |  |  |  |
| Unvaccinated | 477967 (10.4) | 66147 (14.2) | 544114 (10.8) |
| Vaccinated with 1 dose | 127922 (2.8) | 20783 (4.5) | 148705 (2.9) |
| Vaccinated with 2 doses | 3852495 (84.0) | 371939 (80.0) | 4224434 (83.7) |
| Vaccinated with ≥3 doses | 126077 (2.8) | 5796 (1.2) | 131873 (2.6) |

^a^ Continuous variables were presented as mean (± Standard Deviation). Categorical variables were presented as number of individuals and percentages. Standardized differences between the two groups were also presented with 95% confidence interval.

^b^ The sum of counts does not equal the column total because of missing values (≤1%).

**Table S4. Demographic characteristics of participants vaccinated with three and four doses at baseline** ^a^

| **Characteristics** | **Prior infection** | | **Total individuals**  **(N=** **130,248)** |
| --- | --- | --- | --- |
|  | **No**  **(n=** **124,514)** | **Yes**  **(n=** **5,734)** |  |
| Age group, N (%) (years) |  |  |  |
| 12-19 | 970 (0.8) | 33 (0.6) | 1003 (0.8) |
| 20-29 | 9453 (7.5) | 470 (8.1) | 9923 (7.5) |
| 30-39 | 12430 (9.9) | 544 (9.4) | 12974 (9.8) |
| 40-49 | 13754 (10.9) | 665 (11.5) | 14419 (10.9) |
| 50-59 | 18932 (15.0) | 872 (15.0) | 19804 (15.0) |
| 60-69 | 20683 (16.4) | 735 (12.7) | 21418 (16.2) |
| 70-79 | 17955 (14.2) | 677 (11.7) | 18632 (14.1) |
| ≥80 | 31900 (25.3) | 1800 (31.1) | 33700 (25.6) |
| Sex, N (%) |  |  |  |
| Male | 44073 (35.0) | 2028 (35.0) | 46101 (35.0) |
| Female | 82004 (65.0) | 3768 (65.0) | 85772 (65.0) |
| Rural residence, N (%) |  |  |  |
| No | 114756 (91.0) | 5415 (93.4) | 120171 (91.1) |
| Yes | 10689 (8.5) | 332 (5.7) | 11021 (8.4) |
| Neighborhood income quintile ^b^ |  |  |  |
| 1 (lowest) | 20650 (16.4) | 1267 (21.9) | 21917 (16.6) |
| 2 | 22842 (18.1) | 1203 (20.8) | 24045 (18.2) |
| 3 | 22833 (18.1) | 1130 (19.5) | 23963 (18.2) |
| 4 | 25601 (20.3) | 983 (17.0) | 26584 (20.2) |
| 5 (highest) | 33463 (26.5) | 1158 (20) | 34621 (26.3) |
| Public Health Unit ^b^ |  |  |  |
| Central East | 9465 (7.5) | 153 (2.6) | 9618 (7.3) |
| Central West | 24647 (19.5) | 1209 (20.9) | 25856 (19.6) |
| Durham | 6017 (4.8) | 280 (4.8) | 6297 (4.8) |
| Eastern | 7451 (5.9) | 179 (3.1) | 7630 (5.8) |
| North | 6101 (4.8) | 154 (2.7) | 6255 (4.7) |
| Ottawa | 10712 (8.5) | 444 (7.7) | 11156 (8.5) |
| Peel | 6955 (5.5) | 427 (7.4) | 7382 (5.6) |
| South West | 15096 (12.0) | 699 (12.1) | 15795 (12.0) |
| Toronto | 28728 (22.8) | 1666 (28.7) | 30394 (23.0) |
| York | 10273 (8.1) | 536 (9.2) | 10809 (8.2) |
| Number of comorbidities, N (%) |  |  |  |
| 0 | 41806 (33.2) | 1705 (29.4) | 43511 (33.0) |
| 1 | 34823 (27.6) | 1457 (25.1) | 36280 (27.5) |
| 2 | 23339 (18.5) | 1136 (19.6) | 24475 (18.6) |
| ≥3 | 26109 (20.7) | 1498 (25.8) | 27607 (20.9) |
| Essential workers quintile ^b^ |  |  |  |
| 1 (0%–32.5%) | 40026 (31.7) | 1490 (25.7) | 41516 (31.5) |
| 2 (32.5%–42.3%) | 28978 (23.0) | 1339 (23.1) | 30317 (23.0) |
| 3 (42.3%–49.8%) | 23066 (18.3) | 1003 (17.3) | 24069 (18.3) |
| 4 (50.0%–57.5%) | 19119 (15.2) | 1014 (17.5) | 20133 (15.3) |
| 5 (57.5%–100%) | 13890 (11.0) | 864 (14.9) | 14754 (11.2) |
| Persons per dwelling quintile ^b^ |  |  |  |
| 1 (0-2.1) | 32531 (25.8) | 1507 (26.0) | 34038 (25.8) |
| 2 (2.2-2.4) | 22300 (17.7) | 856 (14.8) | 23156 (17.6) |
| 3 (2.5-2.6) | 16921 (13.4) | 718 (12.4) | 17639 (13.4) |
| 4 (2.7-3.0) | 28252 (22.4) | 1302 (22.5) | 29554 (22.4) |
| 5 (3.1-5.7) | 24796 (19.7) | 1317 (22.7) | 26113 (19.8) |
| Uncoupled quintile ^b^ |  |  |  |
| 1 (11.2 - 33.7%) | 25120 (19.9) | 844 (14.6) | 25964 (19.7) |
| 2 (33.7 - 38.4%) | 23198 (18.4) | 885 (15.3) | 24083 (18.3) |
| 3 (38.5 - 43.6%) | 22602 (17.9) | 926 (16.0) | 23528 (17.8) |
| 4 (43.6 - 51.0%) | 24481 (19.4) | 1333 (23.0) | 25814 (19.6) |
| 5 (51.0 - 94.6%) | 29399 (23.3) | 1712 (29.5) | 31111 (23.6) |
| Limited educational attainment quintile ^b^ |  |  |  |
| 1 (0.0-4.1%) | 37792 (30.0) | 1371 (23.7) | 39163 (29.7) |
| 2 (4.1 - 7.5%) | 29834 (23.7) | 1420 (24.5) | 31254 (23.7) |
| 3 (7.5 - 11.4%) | 22328 (17.7) | 998 (17.2) | 23326 (17.7) |
| 4 (11.4 - 17.1%) | 20750 (16.5) | 1054 (18.2) | 21804 (16.5) |
| 5 (17.1 - 94.3%) | 14375 (11.4) | 867 (15.0) | 15242 (11.6) |
| Self-identified visible minority quintile ^b^ |  |  |  |
| 1 (0.0 - 2.2%) | 18305 (14.5) | 566 (9.8) | 18871 (14.3) |
| 2 (2.2 - 7.5%) | 22773 (18.1) | 803 (13.9) | 23576 (17.9) |
| 3 (7.5 - 18.7%) | 28268 (22.4) | 1194 (20.6) | 29462 (22.3) |
| 4 (18.7 - 43.5%) | 31307 (24.8) | 1552 (26.8) | 32859 (24.9) |
| 5 (43.5 - 102%) | 24426 (19.4) | 1595 (27.5) | 26021 (19.7) |
| Recent immigration quintile ^b^ |  |  |  |
| 1-3 (0.0 - 2.1%) | 69263 (54.9) | 2645 (45.6) | 71908 (54.5) |
| 4 (2.1 - 4.7%) | 26842 (21.3) | 1350 (23.3) | 28192 (21.4) |
| 5 (4.7 - 41.2%) | 27989 (22.2) | 1670 (28.8) | 29659 (22.5) |

^a^ Continuous variables were presented as mean (± Standard Deviation). Categorical variables were presented as number of individuals and percentages. Standardized differences between the two groups were also presented with 95% confidence interval.

^b^ The sum of counts does not equal the column total because of missing values (≤1·0%) for this characteristic.

**Table S5. Estimated protection of a past SARS-CoV-2 infection, COVID-19 vaccines, and a hybrid immunity against Omicron-associated hospitalization, stratified by age and time since last infection/vaccination**

Adjusted HRs and their corresponding estimated protection were derived in the multivariable Cox proportional hazards models in the subgroup analysis and adjusted for all covariates listed in appendix p 14 (except for vaccination status). Individuals who were unvaccinated and without a prior infection were served as the reference group. Since a prior SARS-CoV-2 infection was determined by any positive PCR test at least 90 days before an Omicron infection, protection of a prior infection < 3 months since last infection was not applicable. Estimated protection of 4 doses ≥ 3 months was not reported owing to insufficient cases and imprecision in the estimates with wide 95% CIs. In the ≥65 age group, estimated protection of 2 doses and a prior infection at < 3months was not reported owing to insufficient cases and imprecision in the estimates with wide 95% CIs.

|  | **Overall population** | | **≤ 64 years old individuals** | | **≥65 years old individuals** | |
| --- | --- | --- | --- | --- | --- | --- |
| Time since latest antigenic exposure | **Adjusted HR (95%CI)** | **Estimated protection (95%CI)** | **Adjusted HR (95%CI)** | **Estimated protection (95%CI)** | **Adjusted HR (95%CI)** | **Estimated protection (95%CI)** |
| **Unvaccinated** | | |  |  |  |  |
| **Prior infection=0** | **Reference** | **Reference** | **Reference** | **Reference** | **Reference** | **Reference** |
| Prior infection=1 |  |  |  |  |  |  |
| <3 months from last infection^a^ | NA | NA | NA | NA | NA | NA |
| ≥3 months from last infection | 0.33 (0.22, 0.49) | 67% (51%, 78%) | 0.30 (0.19, 0.45) | 70% (55%, 81%) | 0.19 (0.08, 0.45) | 81% (55%, 92%) |
| **Vaccination with 2 doses** | | |  |  |  |  |
| Prior infection=0 |  |  |  |  |  |  |
| ≥0 & <1 months from 2^nd^ dose | 0.64 (0.43, 0.94) | 36% (6%, 57%) | 0.43 (0.25, 0.72) | 57% (28%, 75%) | 1.03 (0.58, 1.80) | -3% (-80%, 42%) |
| ≥1 & <2 months from 2^nd^ dose | 0.42 (0.31, 0.56) | 58% (44%, 69%) | 0.31 (0.22, 0.45) | 69% (55%, 78%) | 0.69 (0.42, 1.12) | 31% (-12%, 58%) |
| ≥2 & <3 months from 2^nd^ dose | 1.11 (0.89, 1.37) | -11% (-37%, 11%) | 0.88 (0.68, 1.13) | 12% (-13%, 32%) | 1.68 (1.18, 2.38) | -68% (-138%, -18%) |
| ≥3 months from 2^nd^ dose | 0.97 (0.90, 1.05) | 3% (-5%, 10%) | 0.46 (0.42, 0.51) | 54% (49%, 58%) | 1.65 (1.49, 1.82) | -65% (-82%, -49%) |
| Prior infection=1 |  |  |  |  |  |  |
| ≥0 & <1 months from last antigenic exposure | NA | NA | NA | NA | NA | NA |
| ≥1 & <2 months from last antigenic exposure | 0.07 (0.01, 0.50) | 93% (50%, 99%) | 0.09 (0.01, 0.59) | 91% (41%, 99%) | NA | NA |
| ≥2 & <3 months from last antigenic exposure | 0.17 (0.04, 0.66) | 83% (34%, 96%) | 0.20 (0.05, 0.80) | 80% (20%, 95%) | NA | NA |
| ≥3 months from last antigenic exposure | 0.17 (0.13, 0.23) | 83% (77%, 87%) | 0.11 (0.08, 0.16) | 89% (84%, 92%) | 0.22 (0.13, 0.36) | 78% (64%, 87%) |
| **Vaccinated with 3 doses** | | | | | | |
| Prior infection=0 |  |  |  |  |  |  |
| ≥0 & <1 months from 3^rd^ dose | 1.97 (1.75, 2.22) | -97% (-122%, -75%) | 1.04 (0.86, 1.26) | -4% (-26%, 14%) | 3.25 (2.84, 3.73) | -225% (-273%,-184%) |
| ≥1 & <2 months from 3^rd^ dose | 0.25 (0.22, 0.29) | 75% (71%, 78%) | 0.11 (0.09, 0.14) | 89% (86%, 91%) | 0.46 (0.40, 0.54) | 54% (46%, 60%) |
| ≥2 & <3 months from 3^rd^ dose | 0.06 (0.05, 0.07) | 94% (93%, 95%) | 0.02 (0.01, 0.02) | 98% (98%, 99%) | 0.11 (0.09, 0.13) | 89% (87%, 91%) |
| ≥3 months from 3^rd^ dose | 0.07 (0.06, 0.09) | 93% (91%, 94%) | 0.03 (0.02, 0.05) | 97% (95%, 98%) | 0.10 (0.08, 0.12) | 90% (88%, 92%) |
| Prior infection=1 |  |  |  |  |  |  |
| ≥0 & <1 months from last antigenic exposure | 0.38 (0.19, 0.75) | 62% (25%, 81%) | 0.43 (0.19, 0.94) | 57% (6%, 81%) | 0.24 (0.06, 0.95) | 76% (5%, 94%) |
| ≥1 & <2 months from last antigenic exposure | 0.03 (0.01, 0.08) | 97% (92%, 99%) | 0.01 (0.00, 0.07) | 99% (93%, 100%) | 0.07 (0.02, 0.20) | 93% (80%, 98%) |
| ≥2 & <3 months from last antigenic exposure | 0.01 (0.00, 0.03) | 99% (97%, 100%) | 0.00 (0.00, 0.03) | 100% (97%, 100%) | 0.03 (0.01, 0.08) | 97% (92%, 99%) |
| ≥3 months from last antigenic exposure | 0.03 (0.01, 0.08) | 97% (92%, 99%) | 0.04 (0.01, 0.15) | 96% (85%, 99%) | 0.01 (0.00, 0.10) | 99% (90%, 100%) |
| **Vaccinated with 4 doses** | | |  |  |  |  |
| Prior infection=0 |  |  |  |  |  |  |
| ≥0 & <1 months from 4^th^ dose | 0.13 (0.08, 0.21) | 87% (79%, 92%) | 100% (100%, 100%) | 100% (100%, 100%) | 0.19 (0.11, 0.31) | 81% (69%, 89%) |
| ≥1 & <2 months from 4^th^ dose | 0.04 (0.02, 0.08) | 96% (92%, 98%) | 0.10 (0.03, 0.40) | 90% (60%, 97%) | 0.05 (0.02, 0.09) | 95% (91%, 98%) |

^a^ A prior SARS-CoV-2 infection was determined by any positive PCR test at least 90 days before an Omicron infection.

**Table S6.** **Estimated protection of a past SARS-CoV-2 infection, COVID-19 vaccines, and a hybrid immunity against Omicron-associated composite severe outcome, stratified by time since last infection/vaccination and age.**

Adjusted HRs and their corresponding estimated protection were derived in the multivariable Cox proportional hazards models in the subgroup analysis and adjusted for all covariates listed in appendix p 14 (except for vaccination status). Individuals who were unvaccinated and without a prior infection were served as the reference group. Since a prior SARS-CoV-2 infection was determined by any positive PCR test at least 90 days before an Omicron infection, protection of a prior infection < 3 months since last infection was not applicable. Estimated protection of 4 doses ≥ 3 months was not reported owing to insufficient cases and imprecision in the estimates with wide 95% CIs. In the ≥65 age group, estimated protection of 2 doses and a prior infection at < 3months was not reported owing to insufficient cases and imprecision in the estimates with wide 95% CIs.

|  | **Overall population** | | **≤ 64 years old individuals** | | **≥65 years old individuals** | |
| --- | --- | --- | --- | --- | --- | --- |
| Time since latest antigenic exposure | **Adjusted HR (95%CI)** | **Estimated protection (95%CI)** | **Adjusted HR (95%CI)** | **Estimated protection (95%CI)** | **Adjusted HR (95%CI)** | **Estimated protection (95%CI)** |
| **Unvaccianted** | | |  |  |  |  |
| **Prior infection=0** | **Reference** | **Reference** | **Reference** | **Reference** | **Reference** | **Reference** |
| Prior infection=1 |  |  |  |  |  |  |
| <3 months from last infection^a^ | NA | NA | NA | NA | NA | NA |
| ≥3 months from last infection | 0.32 (0.22, 0.47) | 68% (53%, 78%) | 0.28 (0.18, 0.43) | 72% (57%, 82%) | 0.18 (0.07, 0.43) | 82% (57%, 93%) |
| **Vaccinated with 2 doses** | | |  |  |  |  |
| Prior infection=0 |  |  |  |  |  |  |
| ≥0 & <1 months from 2^nd^ dose | 1.15 (0.86, 1.54) | -15% (-54%, 14%) | 0.52 (0.33, 0.83) | 48% (17%, 67%) | 2.53 (1.78, 3.59) | -153% (-259%, -78%) |
| ≥1 & <2 months from 2^nd^ dose | 0.45 (0.33, 0.60) | 55% (40%, 67%) | 0.32 (0.22, 0.46) | 68% (54%, 78%) | 0.78 (0.50, 1.21) | 22% (-21%, 50%) |
| ≥2 & <3 months from 2^nd^ dose | 1.15 (0.95, 1.40) | -15% (-40%, 5%) | 0.86 (0.67, 1.11) | 14% (-11%, 33%) | 1.88 (1.35, 2.61) | -88% (-161%, -35%) |
| ≥3 months from 2^nd^ dose | 1.02 (0.94, 1.10) | -2% (-10%, 6%) | 0.48 (0.43, 0.53) | 52% (47%, 57%) | 1.73 (1.57, 1.91) | -73% (-91%, -57%) |
| Prior infection=1 |  |  |  |  |  |  |
| ≥0 & <1 months from last antigenic exposure | 0.34 (0.08, 1.34) | 66% (-34%, 92%) | 0.41 (0.10, 1.62) | 59% (-62%, 90%) | NA | NA |
| ≥1 & <2 months from last antigenic exposure | 0.14 (0.04, 0.56) | 86% (44%, 96%) | 0.16 (0.04, 0.64) | 84% (36%, 96%) | NA | NA |
| ≥2 & <3 months from last antigenic exposure | 0.17 (0.04, 0.66) | 83% (34%, 96%) | 0.19 (0.05, 0.76) | 81% (24%, 95%) | NA | NA |
| ≥3 months from last antigenic exposure | 0.20 (0.15, 0.26) | 80% (74%, 85%) | 0.12 (0.08, 0.17) | 88% (83%, 92%) | 0.29 (0.19, 0.44) | 71% (56%, 81%) |
| **Vaccinated with 3 doses** | | | | | | |
| Prior infection=0 |  |  |  |  |  |  |
| ≥0 & <1 months from 3^rd^ dose | 2.03 (1.81, 2.28) | -103%(-128%, -81%) | 1.06 (0.87, 1.29) | -6% (-29%, 13%) | 3.32 (2.89, 3.80) | -232%(-280%, -189%) |
| ≥1 & <2 months from 3^rd^ dose | 0.28 (0.25, 0.32) | 72% (68%, 75%) | 0.11 (0.09, 0.14) | 89% (86%, 91%) | 0.54 (0.46, 0.63) | 46% (37%, 54%) |
| ≥2 & <3 months from 3^rd^ dose | 0.07 (0.06, 0.08) | 93% (92%, 94%) | 0.02 (0.01, 0.02) | 98% (98%, 99%) | 0.14 (0.12, 0.16) | 86% (84%, 88%) |
| ≥3 months from 3^rd^ dose | 0.10 (0.09, 0.11) | 90% (89%, 91%) | 0.04 (0.03, 0.06) | 96% (94%, 97%) | 0.13 (0.11, 0.16) | 87% (84%, 89%) |
| Prior infection=1 |  |  |  |  |  |  |
| ≥0 & <1 months from last antigenic exposure | 0.49 (0.27, 0.88) | 51% (12%, 73%) | 0.47 (0.22, 0.98) | 53% (2%, 78%) | 0.45 (0.17, 1.19) | 55% (-19%, 83%) |
| ≥1 & <2 months from last antigenic exposure | 0.03 (0.01, 0.07) | 97% (93%, 99%) | 0.01 (0.00, 0.07) | 99% (93%, 100%) | 0.06 (0.02, 0.19) | 94% (81%, 98%) |
| ≥2 & <3 months from last antigenic exposure | 0.01 (0.00, 0.03) | 99% (97%, 100%) | 0.00 (0.00, 0.03) | 100% (97%, 100%) | 0.03 (0.01, 0.08) | 97% (92%, 99%) |
| ≥3 months from last antigenic exposure | 0.04 (0.02, 0.10) | 96% (90%, 98%) | 0.04 (0.01, 0.14) | 96% (86%, 99%) | 0.04 (0.01, 0.13) | 96% (87%, 99%) |
| **Vaccinated with 4 doses** | | |  |  |  |  |
| Prior infection=0 |  |  |  |  |  |  |
| ≥0 & <1 months from 4^th^ dose | 0.17 (0.11, 0.26) | 83% (74%, 89%) | 100% (100%, 100%) | 100% (100%, 100%) | 0.25 (0.16, 0.38) | 75% (62%, 84%) |
| ≥1 & <2 months from 4^th^ dose | 0.04 (0.02, 0.07) | 96% (93%, 98%) | 0.10 (0.02, 0.38) | 90% (62%, 98%) | 0.04 (0.02, 0.08) | 96% (92%, 98%) |

^a^ A prior SARS-CoV-2 infection was determined by any positive PCR test at least 90 days before an Omicron infection.

**REFERENCES**

1. Public Health Ontario. SARS-CoV-2 (COVID-19 Virus) Variant of Concern (VoC) Screening and Genomic Sequencing for Surveillance. 2021 (accessed Feb 1 2022).

2. Statistics Canada/Statistique Canada. Dictionary, Census of Population, 2016. 2020. <https://www12.statcan.gc.ca/census-recensement/2016/ref/dict/geo021-eng.cfm> (accessed October 13 2020).

3. Statistics Canada/Statistique Canada. Questionnaire(s) and Reporting guide(s) - Census 2A-L - 2016. . 2016.

4. Statistics Canada/Statistique Canada. Classification of highest certificate, diploma or degree. 2016. <https://www23.statcan.gc.ca/imdb/p3VD.pl?Function=getVD&TVD=306216> (accessed October 13 2020).

5. Statistics Canada/Statistique Canada. Classification of marital status. 2016. <https://www23.statcan.gc.ca/imdb/p3VD.pl?Function=getVD&TVD=252495> (accessed October 13 2020).

6. Statistics Canada/Statistique Canada. Classification of mobility status, five years. 2016. <https://www23.statcan.gc.ca/imdb/p3VD.pl?Function=getVD&TVD=243793> (accessed October 13 2020).

7. Gershon AS, Wang C, Guan J, Vasilevska-Ristovska J, Cicutto L, To T. Identifying patients with physician-diagnosed asthma in health administrative databases. *Canadian respiratory journal* 2009; **16**(6): 183-8.

8. Gershon A, Wang C, Guan J, Vasilevska-Ristovska J, Cicutto L, To T. Identifying individuals with physcian diagnosed COPD in health administrative databases. *COPD: Journal of Chronic Obstructive Pulmonary Disease* 2009; **6**(5): 388-94.

9. Jaakkimainen RL, Bronskill SE, Tierney MC, et al. Identification of physician-diagnosed Alzheimer’s disease and related dementias in population-based administrative data: a validation study using family physicians’ electronic medical records. *Journal of Alzheimer's Disease* 2016; **54**(1): 337-49.

10. Antoniou T, Zagorski B, Loutfy MR, Strike C, Glazier RH. Validation of case-finding algorithms derived from administrative data for identifying adults living with human immunodeficiency virus infection. *PloS one* 2011; **6**(6): e21748.

11. Tu K, Campbell NR, Chen Z-L, Cauch-Dudek KJ, McAlister FA. Accuracy of administrative databases in identifying patients with hypertension. *Open medicine* 2007; **1**(1): e18.

12. Lipscombe LL, Hwee J, Webster L, Shah BR, Booth GL, Tu K. Identifying diabetes cases from administrative data: a population-based validation study. *BMC health services research* 2018; **18**(1): 1-8.

13. Fleet JL, Dixon SN, Shariff SZ, et al. Detecting chronic kidney disease in population-based administrative databases using an algorithm of hospital encounter and physician claim codes. *BMC nephrology* 2013; **14**(1): 1-8.

14. Schultz SE, Rothwell DM, Chen Z, Tu K. Identifying cases of congestive heart failure from administrative data: a validation study using primary care patient records. *Chronic diseases and injuries in Canada* 2013; **33**(3).

15. Tu JV, Chu A, Donovan LR, et al. The Cardiovascular Health in Ambulatory Care Research Team (CANHEART) using big data to measure and improve cardiovascular health and healthcare services. *Circulation: Cardiovascular Quality and Outcomes* 2015; **8**(2): 204-12.

16. Chung H, Fung K, Ferreira-Legere L, Chen B, Ishiguro L, Kalappa G. COVID- 19 Laboratory Testing in Ontario: Patterns of Testing and Characteristics of Individuals Tested, as of April 30, 2020. Toronto, ON: ICES, 2020.

17. Widdifield J, Bernatsky S, Paterson JM, et al. Accuracy of Canadian health administrative databases in identifying patients with rheumatoid arthritis: a validation study using the medical records of rheumatologists. *Arthritis care & research* 2013; **65**(10): 1582-91.

18. Benchimol EI, Guttmann A, Griffiths AM, et al. Increasing incidence of paediatric inflammatory bowel disease in Ontario, Canada: evidence from health administrative data. *Gut* 2009; **58**(11): 1490-7.

19. Benchimol EI, Guttmann A, Mack DR, et al. Validation of international algorithms to identify adults with inflammatory bowel disease in health administrative data from Ontario, Canada. *Journal of clinical epidemiology* 2014; **67**(8): 887-96.

20. Lapointe-Shaw L, Georgie F, Carlone D, et al. Identifying cirrhosis, decompensated cirrhosis and hepatocellular carcinoma in health administrative data: a validation study. *PLoS One* 2018; **13**(8): e0201120.
